# Author Correction: Population dynamics of head-direction neurons during drift and reorientation

**DOI:** 10.1038/s41586-023-06126-0

**Published:** 2023-05-04

**Authors:** Zaki Ajabi, Alexandra T. Keinath, Xue-Xin Wei, Mark P. Brandon

**Affiliations:** 1 Department of Psychiatry, Douglas Hospital Research Centre, McGill University, Verdun, Quebec Canada; 2 Integrated Program in Neuroscience, McGill University, Montreal, Quebec Canada; 3 Department of Neuroscience, University of Texas at Austin, Austin, TX USA; 4 Department of Psychology, University of Texas at Austin, Austin, TX USA; 5 Center for Perceptual Systems, University of Texas at Austin, Austin, TX USA; 6 Center for Theoretical and Computational Neuroscience, University of Texas at Austin, Austin, TX USA; 7 Present Address: Department of Neurobiology, Harvard Medical School, Boston, MA USA

**Keywords:** Neural circuits, Network models, Spatial memory

Correction to: *Nature* 10.1038/s41586-023-05813-2 Published online 22 March 2023

In the version of this article initially published, there was a typographical error in the seventh sentence of the Methods “Surgeries” subsection, where in the text now reading “One week after injection, a 0.5-mm-diameter gradient refractive index (GRIN) relay lens (Go!Foton) was implanted above the ADN (AP, –1.05; ML, 0.8; DV, −3),” the AP coordinate “–1.05” originally read “1.8.” The error has been corrected in the HTML and PDF versions of the article.

