# Supplementary material for: Population dynamics of head-direction neurons during drift and reorientation
Source: Nature. 2023 Mar 22;615(7954):892–9. doi: 10.1038/s41586-023-05813-2 (PMC10060160; doi:10.1038/s41586-023-05813-2)
Supplement: Supplementary file 1 — Supplementary Figs. 1–6 and Supplementary References. [file 41586_2023_5813_MOESM1_ESM.pdf]

nature portfolio

https://doi.org/10.1038/s41586-023-05813-2

Supplementary information

Population dynamics of head-direction neurons during drift and reorientation

In the format provided by the authors and unedited

Supplementary material: Attractor network model

The goal of our model is to propose a potential neural mechanism of drift control, in the HD system, through gain modulation. The neuronal model is an extension of what was described in Redish et al. (1996)[1] and Redish (1999)[2]. We made important changes to the network design which will be detailed below.

Model inputs:

α_{in}: Time-series of true network gain

AV: Time-series of true head angular velocity (AV), in degrees/frame

θ_{cue}: Angle of current cue on display, in degrees

θ_{HD}: Animal's true head direction, in degrees

D_{ini}: Initial drift offset, in degrees

L: Binary time-series indicating cue display (0:cue-off, 1:cue-on)

N: Number of neurons in the layer

Model outputs:

α_{sim}: Time series of simulated network gain of the HD-neuron layer

D_{sim}: Time series of simulated drift signal, in degrees

1- Network design

Our attractor neural network consists of three layers (pools): (1) The HD layer, (2) the inhibition layer and, (3) the conjunctive AV-by-HD layer which itself can be divided into two sub-layers: (3a) the CW-AV-by-HD layer and (3b) the CCW-AV-by-HD layer. Extra-network input comes in the form of a visual layer, AV cells (CW and CCW) of the vestibular system as well as a global modulation source (i.e., gain cell).

1-1 Generation circuit:

First, we show how the generation circuit (layers (1), (2) and (3)) can produce a stable HD representation. Model figures 1, 2 and 3 show the connections between the layers. Our model can maintain a unique bump of activity (at the HD layer) through constant input from the vestibular system (AV-by-HD layer) combined with lateral inhibition from the inhibitory layer. In this regard, our model falls within the same category as Song & Wang (2005)[3], though clear distinctions can be identified between the two models. Our choice of departing from standard HD-network models that use recurrent excitation at the HD layer to generate a unique bump of activity is motivated by anatomical and physiological studies of the HD circuitry, in rodents. We know that two types of AV cells exist in the vestibular system: symmetric and asymmetric AV cells (CW-AV cells and CCW-AV cells)[4,5]. While the symmetric AV cells increase their firing rate proportional to the head AV, regardless of direction, the asymmetric AV cells' activity increases only in one direction and decreases in the other. Interestingly, both asymmetric AV cells' subtypes (CW and CCW) appear to fire at higher rates than minimum values, albeit with equal amounts, when the animal's head is not moving. We also know the involvement of inhibition in the generation of HD-cell activity from previous studies that showed the connections in the downstream pathway of the generation circuit (DTN→LMN) are largely GABAergic[6,7]. Additionally, there is no anatomical evidence for the existence of recurrent excitation neither in the generation circuit (DTN and LMN) nor in the thalamus (ADN). Therefore, achieving a stable representation by combining the activity from the vestibular system together with a lateral inhibition (as in the current model) appears more biologically plausible.

Neuronal dynamics

We use an approach similar to Redish et al. (1996)[1] to model the firing activity of every neuron in the HD, inhibitory and AV-by-HD layers. Each postsynaptic unit's response is governed by three equations:

$$V_i(t) = \gamma_i + \sum_j w_{ij} S_j(t) \tag{1}$$

$$F_i(t) = a\ log(1 + e^{V_i(t)-b}) \tag{2}$$

$$\tau_i \frac{dS_i(t)}{dt} = -S_i(t) + F_i(t) \tag{3}$$

Where, V_i is the voltage of postsynaptic neuron i, γ_i is the tonic inhibition term, $\omega_{i,j}$ is the synaptic weight between postsynaptic neuron i and presynaptic neuron j, S_j is the synaptic drive of presynaptic neuron j, F_i is the activation function of postsynaptic neuron i, dt is the time step and, τ_i is the time constant defining the decay rate of postsynaptic potential (PSP). Equations (1) and (3) follow directly from Redish et al. (1996)[1]. However, we opted for a more biologically plausible activation function F_i, in (2), following Zhang (1996)[8] which also has the advantage over the hyperbolic tangent (used in Redish et al. (1996)[1]) of preventing saturation issues at high activity levels. Both a and b, in (2), are optimization parameters that control the scale and shift of the activation function, respectively.

Our model is composed of the same number of units (neurons), N, per layer/sublayer. To understand how this works, it is useful to divide the horizontal plane into N equally distant directions which we will refer to as preferred firing directions (PFDs). We, then, assign to every unit, in each layer/sublayer, a unique PFD. We refer to neurons with the same PFD but belonging to different layers as 'counterparts'.

Interactions between the HD and the inhibitory layers:

Each inhibitory unit sends projections to all HD neurons. To ensure the maintenance of a bell-shaped activity packet at the HD layer, the synaptic weights are determined by a Gaussian kernel such that they become stronger with the increase in PFD distance between the inhibitory neuron and its target HD neuron. Effectively, when active, an inhibitory neuron causes minimal decrease in firing activity within a close neighborhood of its counterpart HD neuron while it engenders maximal inhibition on distant HD units.

The connection weight of the projection from inhibitory neuron i onto HD neuron j is, thus, given by:

$$w_{I\to HD}(\phi_i^I, \phi_j^{HD}) = k_I(e^{\frac{-(\phi_i^I-\phi_j^{HD})^2}{\sigma_I{}^2}} - 1) \quad (4)$$

Where, φ_i^I and φ_i^{HD} are PFDs of inhibitory neuron i and HD neuron j, respectively, k_I is a scale factor and, σ_I is the standard deviation of the weight distribution.

Meanwhile, each inhibitory neuron receives an excitatory back projection from its unique counterpart HD neuron. This ensures only a subset of the inhibitory pool (counterpart of the activity packet) is active, at any given time, resulting in lateral inhibition of HD neurons outside the activity packet.

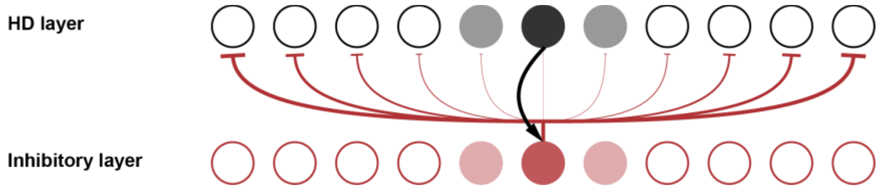

Model Figure 1. Connections between the HD layer and the inhibitory layer. Triangular arrowhead indicates excitatory projection. Flat arrowhead indicates inhibitory projection. Color gradients indicate the level of activity for each neuron (i.e., opacity increases with firing activity). Arrow thickness indicates synaptic strength (i.e., thickness increases with synaptic weight). For clarity, we only show projections from the unit with highest activity, at each layer

Interactions between the HD and the AV-by-HD layers:

Similar to the inhibitory layer, every AV-by-HD neuron sends direct projections to all HD neurons. The synaptic weights are determined by a Gaussian kernel that peaks at an offset location w.r.t the counterpart HD neuron. That is, an AV-by-HD unit provides the highest excitation either rightwards (for a CW-AV-by-HD neuron) or leftwards (for a CCW-AV-by-HD neuron) of the counterpart HD neuron. This configuration has the advantage of allowing more flexibility and fine tuning in the calibration of the system (i.e., matching vestibular input to visual flow). The amount of offset is kept to be the same (in absolute value terms) between

CW and CCW AV-by-HD units. This ensures balanced input from both sublayers when the animal's head is not rotating.

The connection weight of the projection from AV-by-HD neuron i onto HD neuron j is given by:

$$w_{AVxHD\rightarrow HD}\left(\phi_i^{AVxHD},\phi_j^{HD}\right) = k_{AVxHD}e^{\frac{-(\phi_i^{AVxHD}-(\phi_j^{HD}\pm\varphi_{offset})^2}{\sigma_{AVxHD}{}^2}} \quad (5)$$

Where, φ_i^{AVxHD} and φ_i^{HD} are PFDs of AV-by-HD neuron i and HD neuron j, respectively, k_{AVxHD} is a scale factor, σ_{AVxHD} is the standard deviation of the weight distribution and, ϕ_{offset} is the angular offset between AV-by-HD and HD layers. Meanwhile, each AV-by-HD neuron receives an excitatory back projection from its unique counterpart HD neuron. This ensures only a subset of the AV-by-HD pool (counterpart of the activity packet) has higher activity rates than the rest, at any given time.

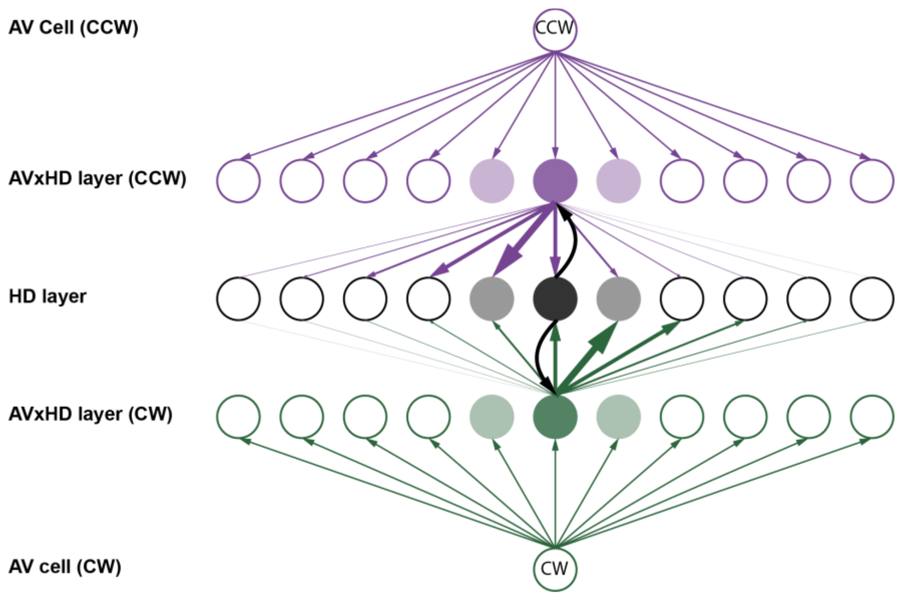

Model Figure 2. Connections between the HD layer and the AV-by-HD layers. Triangular arrowhead indicates excitatory projection. Color gradients indicate the level of activity for each neuron (i.e., opacity increases with firing activity). Arrow thickness indicates synaptic strength (i.e., thickness increases with synaptic weight). For clarity, we only show projections from the unit with highest activity, at each layer

Voltage calculation:

Putting together, we can write the voltage equations for every neuron, in each layer, as the following:

$$V_i^{HD-gen}(t) = \gamma_{HD} + \sum_j w_{I\to HD}(\phi_j, \phi_i) S_j^I(t) + \sum_j w_{CW\to HD}(\phi_j, \phi_i) S_j^{CW}(t)$$
$$+ \sum_j w_{CCW\to HD}(\phi_j, \phi_i) S_j^{CCW}(t) \qquad (6)$$

$$V_i^I(t) = \gamma_I + \sum_j w_{HD\to I} S_j^{HD}(t) \qquad (7)$$

$$V_i^{CW}(t) = \gamma_{CW} + AV^{CW}(t) + \sum_j w_{HD\to CW} S_j^{CW}(t) \qquad (8')$$

$$V_i^{CCW}(t) = \gamma_{CCW} + AV^{CCW}(t) + \sum_j w_{HD\to CCW} S_j^{CCW}(t) \qquad (8'')$$

Where, V_i^{HD-gen} is a unit's voltage in the HD layer, considering the generation circuit only, V_i^I is a unit's voltage in the inhibitory layer and, V_i^{CW} and V_i^{CCW} are units' voltages in CW-AV-by-HD and CCW-AV-by-HD layers, respectively. AV^{CW} and AV^{CCW} are excitatory inputs reflecting the activity of asymmetric AV cells such that, at any given time:

$$AV^{CW}(t) = \frac{1}{AV^{CCW}(t)} \qquad (9)$$

When the animal's head is not rotating, the HD network receives equal excitation from both AV cell types such that $AV^{CW}(t) = AV^{CCW}(t) = 1$

1-2 Visual control of the HD network via gain modulation:

Visual input:

The visual input is provided by an extra network layer of N neurons, each of which provides an excitatory input to its counterpart HD neuron. When the visual input is available, activity on the visual layer is defined by a Gaussian kernel such that

$$V_i^{Vis}(t) = k_{Vis} e^{\frac{(\phi_{Vis,i} - \theta_{Vis}(t))^2}{-2\sigma_{Vis}{}^2}} \qquad (10)$$

where, $\phi_{Vis,i}$ is the preferred firing direction of visual neuron i, σ_{Vis} is the standard deviation of the visual Gaussian kernel and, $\theta_{Vis}(t)$ is the animal's head direction, at time t, w.r.t the visual reference frame, such that: $\theta_{Vis}(t) = \theta_{HD}(t) - \theta_{cue}(t)$. The input is multiplied by a constant k_{Vis}.

Gain modulation:

We model gain modulation as an extra-network input g that affects uniformly all HD neurons, which takes the form of an affine function of the experimental gain $\alpha_{in}(t)$, such that:

$$g(t) = k_g(\alpha_{in}(t) - 1) \qquad (11)$$

where, k_g is a positive constant. This input can be either excitatory ($\alpha_{in}(t) > 1$) or inhibitory ($\alpha_{in}(t) < 1$).

Integration of visual input and gain modulation

Both the visual and the gain modulation inputs constitute the downstream input to the HD layer. The final form of the voltage at a given HD neuron thus becomes:

$$V_i^{HD}(t) = V_i^{HD-gen}(t) + V_i^{Vis}(t)L + g(t) \qquad (12)$$

where L indicates whether the visual cue is available. It has been demonstrated, in Jackson & Redish (2003)[9], that adding an extra-network excitatory input (equivalent to the visual input, in our case) causes the bump of activity to shift towards the direction of highest external excitation and realigns the current HD representation with the location of the external input, regardless of any movement of the animal's head. Depending on the strength of this input, as well as its distance to the current representation, the bump can either rotate continuously and span all intermediate directions (i.e., when the external input is near the current representation) or jump abruptly to the new location (i.e., when the external input is far away from the current representation). Our model allows rotations at different speeds to occur regardless of the visual

input's strength or distance to the current representation, which better reflects our experimental data. This can be achieved through gain modulation. For example, when the g is negative (i.e., inhibitory), the activity on the HD layer decreases which results in a weaker lateral inhibition. This makes it easier for an external input to activate HD neurons outside the current activity packet to cause a shift in representation (note that this is a winner-takes-all situation which means, at any given time, there can only be a unique bump of activity). Conversely, if g is positive (i.e., excitatory), the increased activity on the HD layer renders the activation of HD neurons outside the activity packet harder and would need a stronger external input than in the previous case (i.e., $g < 0$) to achieve a similar shift in representation.

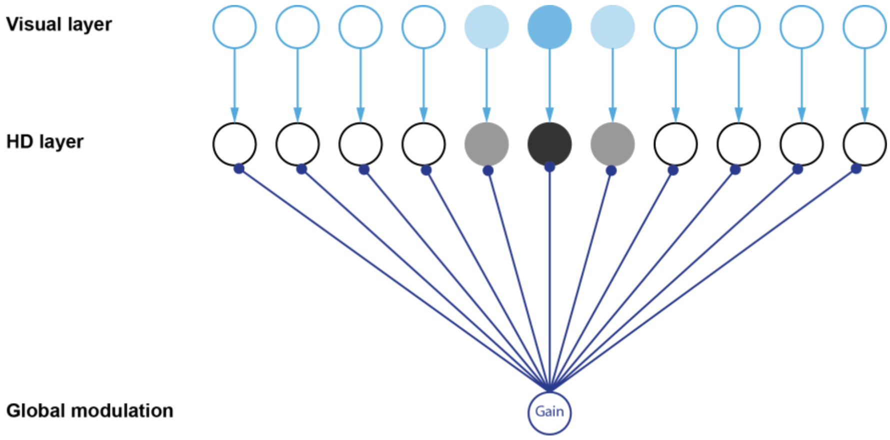

Model Figure 3. Visual control and gain modulation of the HD layer. Triangular arrowhead indicates excitatory projection. Round arrowhead indicates projection that can be excitatory (i.e., $g > 0$) as well as inhibitory (i.e., $g < 0$). Color gradients indicate the level of activity for each neuron (i.e., opacity increases with firing activity). Arrow thickness indicates synaptic strength (in this case, all synapses have similar weights).

2- Simulation of drift and output gain

Once all variables have been defined, we can simulate different reset scenarios by displaying the cue at different positions while varying the input gain using experimental data. For simplicity, we assume a noise-free model. To estimate the internal HD representation, we identify the peak location of the synaptic drive on the HD layer such that:

$$\tau_i \frac{dS_i^{HD}(t)}{dt} = -S_i^{HD}(t) + F_i^{HD}(t) \quad (13)$$

$$\theta_{sim}^{HD}(t) = \underset{\phi_i^{HD}}{argmax}\, S^{HD}(t) \qquad (14)$$

where $S^{HD}(t)$ is a Nx1 vector. To calculate the drift, D_{sim}, we simulate two networks in parallel, starting with the same initial conditions. The first network assumes a perfect integration of the vestibular input and does not include any visual interference or gain modulation ($g = 0$). This constitutes the reference HD, θ_{ref}^{HD} (equivalent to the measured HD, in our experimental data). The second network includes the visual input, as well as the gain modulation as described in paragraph 2-2. From the second network, we obtain θ_{sim}^{HD}. The simulated drift is simply the angular difference between the two quantities:

$$D_{sim}(t) = \theta_{ref}^{HD}(t) - \theta_{sim}^{HD}(t) \qquad (15)$$

To calculate the output gain, α_{sim}, we use the egocentric version of S^{HD}, defined as:

$$S_{ego}^{HD}(t) = circshift(S^{HD}(t), -\theta_{sim}^{HD}(t)) \qquad (16)$$

where $circshift$ represents the circular shift operator. This has the effect of bringing the activity packet to the center of the internal HD space (see example, in main text, Fig. 2E). Then, we perform a linear regression such that:

$$S_{ego,BL}^{HD}\alpha_{sim}(t) = S_{ego}^{HD}(t) \qquad (17)$$

where $S_{ego,BL}^{HD}$ is a Nx1 vector corresponding to the egocentric synaptic drive profile of the HD layer, in baseline simulation.

3- Optimization

The goal of the optimization is to determine the parameter values that minimize the classification error of the drift signal (i.e., fast vs slow) while ensuring the output gain does not deviate from the input gain. Note that the output gain is not guaranteed to be the same as the input gain, due to the complex interactions between the multiple layers and the intrinsic firing properties of each unit, in the network.

We define the distance between output drift and the mean input drift, for both fast and slow resets, as follows:

$$Err_{drift}^{fast}(i) = \sum_t (D_{sim,t}^{i} - D_{ref,t}^{fast})^2 \qquad (18)$$

$$Err_{drift}^{slow}(i) = \sum_t (D_{sim,t}^{i} - D_{ref,t}^{slow})^2 \qquad (19)$$

where $D_{ref,t}^{fast}$ is the mean drift signal for fast-reset examples of the training set and $D_{ref,t}^{slow}$ is the mean drift signal for slow-reset examples of the training set.

We then determine the simulated reset type of example i such that:

$$\begin{cases} Reset_{Sim}(i) = 1, if Err_{drift}^{fast}(i) < Err_{drift}^{slow}(i) \\ Reset_{Sim}(i) = 2, if Err_{drift}^{fast}(i) > Err_{drift}^{slow}(i) \end{cases} \qquad (20)$$

Similarly, we define the distance between output and input gains:

$$Err_{gain}(i) = \sum_t (\alpha_{sim,t}^{i} - \alpha_{in,t}^{i})^2 \qquad (21)$$

Finally, we use a search algorithm to find the optimal parameters $\hat{\mathcal{P}}$ such that:

$$\hat{\mathcal{P}} = \underset{\mathcal{P}}{argmin}(\sum_i \left(Reset_{Sim}(i) - Reset_{Ref}(i)\right)^2 + c_{opt} Err_{gain}(i)) \qquad (22)$$

where P is the set of parameters to be optimized, $Reset_{Ref}$ is a 1-D vector indicating the type of the training reset examples (i.e., 1=fast and 2=slow), and c_{opt} is a penalizing term that determines how much weight is attributed to the minimization of input-output gain error.

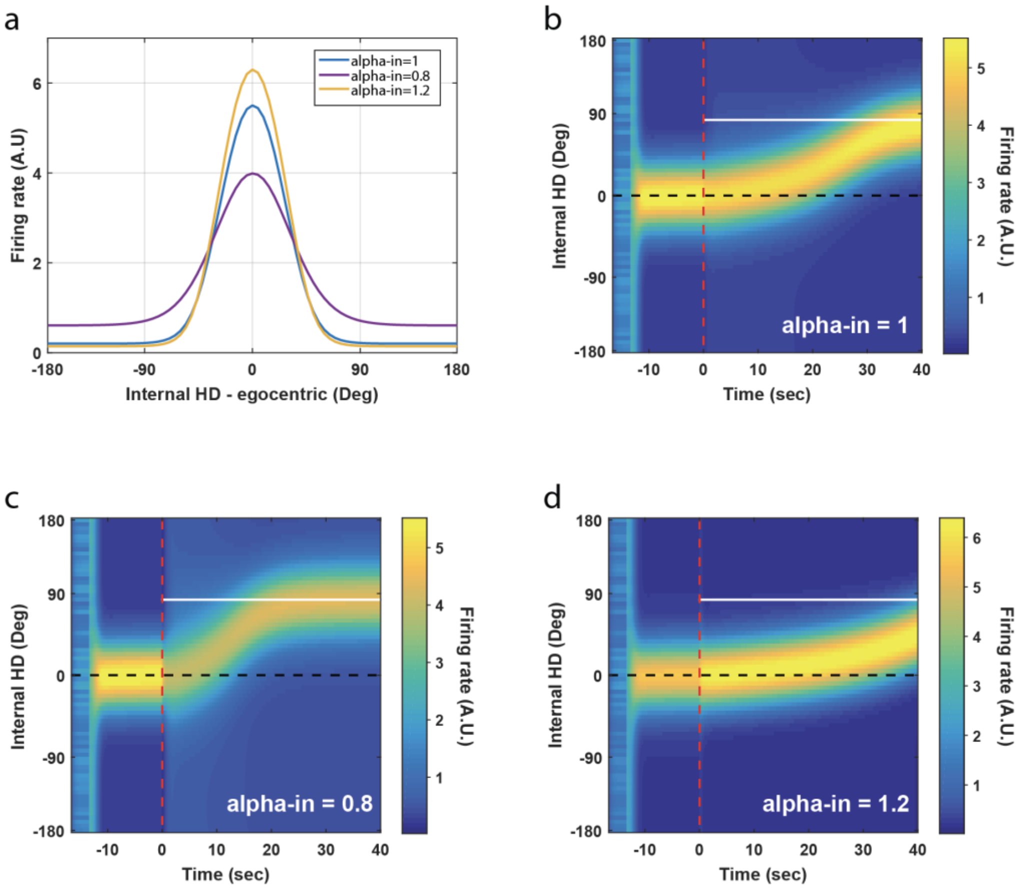

Model Figure 4. Simulation results showing modulation of reset speed at different gain amplitudes (i.e. "alpha-in"). a. Due to weakening lateral inhibition in the HD network, at low gain amplitudes, the egocentric bump of activity exhibits widening of the tuning curve as well as an increase in baseline firing rate. b, c and d. Time-dependent dynamics of the bump of activity before and after cue-display (dashed red line). The first 5 seconds ([20:15]s pre-cue) correspond to a random cell activity, after which an external input is applied on the network to impose the formation of the bump of activity at a specific direction (0° in this case). During the pre-cue period, alpha-in is set to 1. Following cue display, alpha-in changes instantaneously and remains at the indicated value for the remainder of the simulation. The 40s post-cue simulation interval may or may not be sufficient to shift and stabilize the network's HD representation around the visual cue location (solid white line), depending on the gain amplitude. In all these examples, the animal is motionless (Angular velocity = 0°/s).

Model Table 1: Simulation parameters

dt	Time step	30ms
N	Number of neurons per layer (HD, inhibitory, AV-by-HD and visual layers)	75
τ_i^{HD}	Time constant of the decay rate of postsynaptic potential for HD neurons	300ms
τ_i^{I}	Time constant of the decay rate of postsynaptic potential for inhibitory neurons	60ms
$\tau_i^{AV-by-HD}$	Time constant of the decay rate of postsynaptic potential for AV-by-HD neurons	60ms
γ_i^{HD}	Tonic inhibition of HD neurons	-1.5
γ_i^{I}	Tonic inhibition of inhibitory neurons	-7.5
$\gamma_i^{AV-by-HD}$	Tonic inhibition of AV-by-HD neurons	-1
a	Scale parameter of the activation function F_i	1.1
b	Shift parameter of the activation function F_i	0.25
$w_{HD \to I}$	HD → I connection weights	3
$w_{HD \to AV-by-H}$	HD → AV-by-HD connection weights	0.3
ϕ_{offset}	Angular offset of AV-by-HD → HD projections	24°
k_{Vis}	Scale parameter of visual input strength	0.32
σ_{Vis}	Standard deviation of the visual input kernel	11°
k_g	Scale parameter of input gain	4.8
k_{AVxHD}	Scale parameter of AV-by-HD → HD connection	1.6248
σ_{AVxHD}	Standard deviation of AV-by-HD → HD weight distribution	5°
k_I	Scale parameter of I → HD connection	0.0432
σ_I	Standard deviation of I → HD weight distribution	15°

4- Plasticity

Our experimental data demonstrated that the duration of an animal's exposure to a shifted-cue context predicted the drift behavior of the internal HD representation, in a subsequent darkness. The time-dependent nature of these observations led us to hypothesize that plasticity could explain the variability in representational drift patterns. Previous theoretical works have already speculated about plasticity involvement in the HD network[10-13]. Indeed, computational models show that, through Hebbian learning, long-term associations between HD neurons and 'landmark cells' – which convey information about sensory cues – could form, thus leading to a successful integration of combined self-motion cues and sensory perception of landmarks, and a stable internal representation of the directional experience within the environment. Though there is no direct experimental evidence for plasticity in the mammalian HD system, research in fruit flies[13,14] has provided key insights into the neural mechanisms that underlie the formation of stable internal HD representations and convincing evidence for the involvement of plastic processes in the HD network. Specifically, a type of cells called 'ring neurons'[13-15] may fit the role of 'landmark cells'. Each of these cells synapses onto all compass neurons (equivalent to HD cells in mammals). More so, these synapses are suggested to be plastic which allows the formation of long-term associations between compass neurons and visual scenes.

In our model, we propose an additional layer that we refer to as the 'sensorimotor-by-HD' layer and composed of neurons that are akin to ring neurons. Each sensorimotor-by-HD cell synapses onto all neurons of the HD layer via plastic synapses (Model Fig. 5). Through Hebbian learning, new associations can be formed between the two layers depending on the animal's experience within the environment. The sensorimotor-by-HD layer reflects a consensus about the current sensory experience, and we hypothesize that it can only sustain

movements of a unique bump of activity. This means that the emergence of activity on this layer is likely mediated by a (cortical) attractor network that operates in concert with the main attractor network. However, this hypothesis is out of the scope of this work.

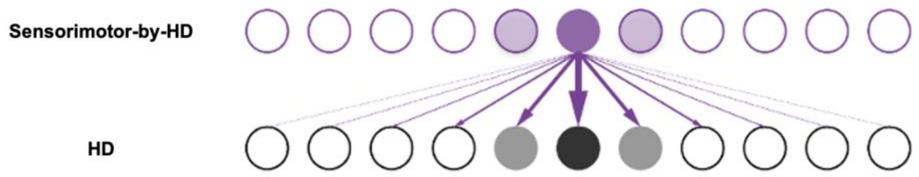

Model Figure 5. Connections between the Sensorimotor-by-HD and HD layers. Projections from the most active sensorimotor-by-HD neuron only are shown. Arrows represent plastic synapses that are updated following a Hebbian rule. Width of arrows indicates synaptic strength. All shown connections are excitatory. Color gradients indicate the level of activity for each neuron (i.e., opacity increases with firing activity).

To implement plasticity in the model, we applied a Hebbian update rule – following Skaggs et al. (1994)[16] – on synaptic weights linking each sensorimotor-by-HD cell to all HD neurons, such that:

$$\Delta w_{i\to j}(t) = \alpha(w_{max} f_H\left(r^j(t)\right) - w_{i\to j}(t))r^i(t) \qquad (23)$$

Where, $\Delta w_{i\to j}$ is the change in synaptic weight from presynaptic sensorimotor-by-HD neuron i to postsynaptic HD neuron j, α is the learning rate, w_{max} is the upper bound of synaptic weights, $w_{i\to j}$ is the synaptic weight from presynaptic sensorimotor-by-HD neuron i to postsynaptic HD neuron j, r^i and $r^j$are the firing rates of presynaptic sensorimotor-by-HD neuron i and postsynaptic HD neuron j, respectively. f_H is a sigmoidal activation function of the form:

$$f_H(x) = \frac{c_H}{1+e^{b_H - a_H x}} \qquad (24)$$

Where, $a_H, b_H and c_H$are optimization parameters. After each iteration, the synaptic weights are updated as follows:

$$w_{i\to j}(t) \leftarrow w_{i\to j}(t) + \Delta w_{i\to j}(t) \qquad (25)$$

Which we integrate in the total voltage of each neuron of the HD layer such that:

$$V^{HD-Hebbian}(t) = V^{HD}(t) + g_H W_{sm-by-HD \to HD} V^{sm-by-HD}(t) \quad (26)$$

Where, $V^{HD-Hebbian}$ is a Nx1 vector of HD neuron voltages, V^{HD} is a Nx1 vector of HD neuron voltages excluding the input from the sensorimotor-by-HD layer (see equation 12), g_H is a scaling factor, $W_{sm-by-HD \to HD}$ is a NxN synaptic weight matrix linking the sensorimotor-by-HD and HD layers, and $V^{sm-by-HD}$ is a Nx1 vector of sensorimotor-by-HD neuron voltages. The activity on the sensorimotor-by-HD layer is assumed to have the same shape and amplitude as the visual layer and is updated through circular shifts by assuming a perfect integration of the vestibular input (i.e. with same rate of change of θ_{ref}^{HD}).

Upon optimization, the parameters are assigned the values $\alpha = 40$, $a_H = 20$, $b_H = 15$, $c_H = 2$, and $g_H = 0.8$.

5- Vestibular input recalibration through visual feedback

Our third experiment showed that the visual experience (i.e. continuous cue rotation) can cause a persistent bias in the internal HD representation. We built upon the model in the previous section to gain further insights into the mechanisms that could lead to such behavior in the network.

To account for a flexible integration of the vestibular input, we added a recalibration circuit that adjusts the firing activity of angular velocity (AV) cells depending on external sensory inputs. Our experimental data showed that the internal HD is phase-locked to a rotating visual cue, even when the animal is motionless. The same experiment showed that the representational drift maintains an angular speed similar to the cue's, in a subsequent darkness period. We interpret this observation as an asymmetric bias added unilaterally to the vestibular AV cells (CW or CCW). The resulting imbalance causes movements along the attractor network in the direction of increased bias, even in periods of immobility. We hypothesize that

the bias signal reflects the difference between the ‘perceived’ AV and the ‘vestibular’ AV. We believe the perceived AV is a cortical construct that measures the rate of angular displacement of the bump of activity along the HD layer. Note that the bump’s movement is not only dependent on vestibular and sensorimotor input but also can be affected by plasticity as we saw, in the previous section.

To achieve such calibration, we propose a simple circuit composed of a bias cell that takes as inputs the perceived AV (excitatory) and the vestibular AV (inhibitory) and, through a recurrent excitation (i.e. it synapses onto itself with an excitatory projection), the cell can maintain firing activity that is proportional to the difference in AV inputs (only when the perceived AV is larger than the vestibular AV). The bias cell also synapses onto the vestibular AV neuron through excitatory projections which allows compensation of the vestibular input to match the perceive AV. This circuit

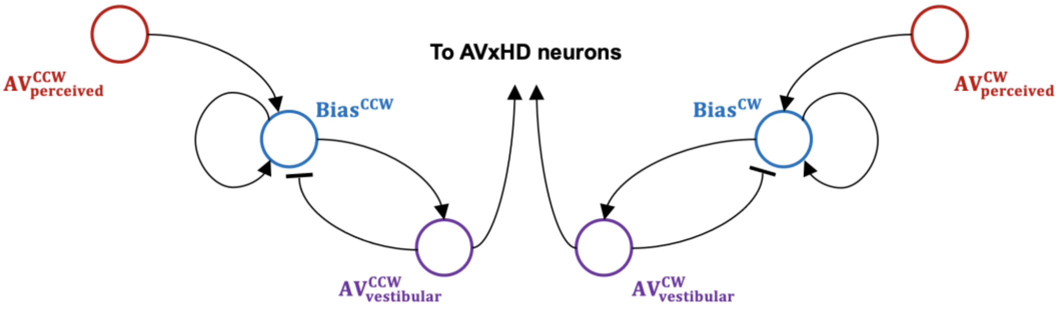

Model Figure 6. Proposed neural circuit for vestibular input recalibration through bias. Triangular arrowheads indicate excitatory projections. Flat arrowheads indicate inhibitory projection.

has a directional specificity (CW or CCW) and is duplicated to allow readjustment of CW and CCW vestibular inputs separately (Model Fig. 6).

To integrate the recalibration circuit into our model, we first update the output signal of the Bias neurons every timestep dt as follows:

$$Bias^{CW}(t+dt) = g_{AV}\left(AV^{CW}_{perceived}(t) - \left(AV^{CW}_{Vestibular}(t) + \beta Bias^{CW}(t)\right)\right)$$

$$+W_{Bcw\to Bcw}Bias^{CW}(t) \qquad (27)$$

$$Bias^{CCW}(t+dt) = g_{AV}\left(AV^{CCW}_{perceived}(t) - \left(AV^{CCW}_{Vestibular}(t) + \beta Bias^{CCW}(t)\right)\right)$$

$$+W_{Bccw\to Bccw}Bias^{CCW}(t) \qquad (28)$$

where g_{AV} is the synaptic weight of the perceived and vestibular AV neurons 'projections onto the bias cell, β represents the synaptic weight of the excitatory projections from the bias neuron onto the vestibular AV neuron and, $W_{Bcw\to Bcw}$ and $W_{Bccw\to Bccw}$ are the synaptic weights of the recurrent projections of CW and CCW bias neurons, respectively. We ensure that the output of these bias cells is always positive by imposing:

$$Bias^{CW}(t) \leftarrow max(0, Bias^{CW}(t)) \qquad (29)$$

$$Bias^{CCW}(t) \leftarrow max(0, Bias^{CCW}(t)) \qquad (30)$$

While we do not have access to the perceived-AV cells, we may still derive their firing rates by assuming that they are proportional to the speed of rotation of the HD layer's bump of activity $\dot{\theta}^{HD}_{sim}$. To allow the bias cells to compare between equivalent AV inputs, the vestibular AV cells 'firing rates are also derived from the speed of rotation of the HD layer's bump of activity in a network that assumes perfect integration of the vestibular input $\dot{\theta}^{HD}_{ref}$ (see section '*2- Simulation of drift and output gain*'):

$$\begin{aligned} AV^{CW}_{perceived}(t) &= k_{AV}H(\dot{\theta}^{HD}_{sim}(t)) \\ AV^{CCW}_{perceived}(t) &= k_{AV}H\left(-\dot{\theta}^{HD}_{sim}(t)\right) \\ AV^{CW}_{vestibular}(t) &= k_{AV}H(\dot{\theta}^{HD}_{ref}(t)) \\ AV^{CCW}_{vestibular}(t) &= k_{AV}H(-\dot{\theta}^{HD}_{ref}(t)) \end{aligned} \qquad (31)$$

Where, k_{AV} is a scaling factor and H is a function defined as:

$$H(x) = \begin{cases} x, x > 0, \\ 0, x \leq 0, \end{cases} \qquad (32)$$

Linking the perceived and vestibular AV cells' firing rates to the bump of activity's speed of rotation means that their activity is highly dependent on the angular resolution of the

network (i.e. number and distribution of HD states (angles) that can be represented) which could limit the network's capability of seamless recalibration across different cue rotation speeds if the resolution is low.

Finally, we modify the AV input to the AV-by-HD cells as follows:

$$AV_{input}^{CW}(t) = AV^{CW}(t) + \beta Bias^{CW}(t) \qquad (33)$$

$$AV_{input}^{CCW}(t) = AV^{CCW}(t) + \beta Bias^{CCW}(t) \qquad (34)$$

where AV^{CW} and AV^{CCW} are the same as in equation (9).

Upon optimization, the optimal parameter values are: $\beta = 0.434$, $g_{AV} = 1$, $W_{Bcw \to Bcw} = 0.1$ and $k_{AV} = 1$.

References

1 Redish, A. D., Elga, A. N. & Touretzky, D. S. A coupled attractor model of the rodent head direction system. *Network: Computation in Neural Systems* **7**, 671-685, doi:10.1088/0954-898X_7_4_004 (1996).

2 Redish, A. D. *Beyond the cognitive map: From place cells to episodic memory*. (The MIT Press, 1999).

3 Song, P. & Wang, X. J. Angular path integration by moving "hill of activity": a spiking neuron model without recurrent excitation of the head-direction system. *J Neurosci* **25**, 1002-1014, doi:10.1523/JNEUROSCI.4172-04.2005 (2005).

4 Clark, B. J. & Taube, J. S. Vestibular and attractor network basis of the head direction cell signal in subcortical circuits. *Front Neural Circuits* **6**, 7, doi:10.3389/fncir.2012.00007 (2012).

5 Bassett, J. P. & Taube, J. S. Neural correlates for angular head velocity in the rat dorsal tegmental nucleus. *J Neurosci* **21**, 5740-5751 (2001).

6 Gonzalo-Ruiz, A., Sanz-Anquela, J. M. & Spencer, R. F. Immunohistochemical localization of GABA in the mammillary complex of the rat. *Neuroscience* **54**, 143-156, doi:10.1016/0306-4522(93)90390-2 (1993).

7 Wirtshafter, D. & Stratford, T. R. Evidence for GABAergic projections from the tegmental nuclei of Gudden to the mammillary body in the rat. *Brain Res* **630**, 188-194, doi:10.1016/0006-8993(93)90656-8 (1993).

8 Zhang, K. Representation of spatial orientation by the intrinsic dynamics of the head-direction cell ensemble: a theory. *J Neurosci* **16**, 2112-2126, doi:10.1523/JNEUROSCI.16-06-02112.1996 (1996).

9 Jackson, J. C. & Redish, A. D. Detecting dynamical changes within a simulated neural ensemble using a measure of representational quality. *Network* **14**, 629-645 (2003).

10 Cope, A. J., Sabo, C., Vasilaki, E., Barron, A. B. & Marshall, J. A. A computational model of the integration of landmarks and motion in the insect central complex. *PLoS One* **12**, e0172325, doi:10.1371/journal.pone.0172325 (2017).

11 Page, H. J. I. & Jeffery, K. J. Landmark-Based Updating of the Head Direction System by Retrosplenial Cortex: A Computational Model. *Front Cell Neurosci* **12**, 191, doi:10.3389/fncel.2018.00191 (2018).

12 Ocko, S. A., Hardcastle, K., Giocomo, L. M. & Ganguli, S. Emergent elasticity in the neural code for space. *Proc Natl Acad Sci U S A* **115**, E11798-E11806, doi:10.1073/pnas.1805959115 (2018).

13 Kim, S. S., Hermundstad, A. M., Romani, S., Abbott, L. F. & Jayaraman, V. Generation of stable heading representations in diverse visual scenes. *Nature* **576**, 126-131, doi:10.1038/s41586-019-1767-1 (2019).

14 Fisher, Y. E., Lu, J., D'Alessandro, I. & Wilson, R. I. Sensorimotor experience remaps visual input to a heading-direction network. *Nature* **576**, 121-125, doi:10.1038/s41586-019-1772-4 (2019).

15 Hulse, B. K. & Jayaraman, V. Mechanisms Underlying the Neural Computation of Head Direction. *Annu Rev Neurosci* **43**, 31-54, doi:10.1146/annurev-neuro-072116-031516 (2020).

16 Skaggs, W. E., Knierim, J. J., Kudrimoti, H. S. & McNaughton, B. L. A model of the neural basis of the rat's sense of direction. *Adv Neural Inf Process Syst* **7**, 173-180 (1995).
