# Supplementary material for: Population dynamics of head-direction neurons during drift and reorientation
Source: Nature. 2023 Mar 22;615(7954):892–9. doi: 10.1038/s41586-023-05813-2 (PMC10060160; doi:10.1038/s41586-023-05813-2)
Supplement: Supplementary file 2 — Reporting Summary [file 41586_2023_5813_MOESM2_ESM.pdf]

nature portfolio

Corresponding author(s): Mark P. Brandon, Zaki Ajabi

Last updated by author(s): Dec 30, 2022

Reporting Summary

Nature Portfolio wishes to improve the reproducibility of the work that we publish. This form provides structure for consistency and transparency in reporting. For further information on Nature Portfolio policies, see our Editorial Policies and the Editorial Policy Checklist.

Statistics

For all statistical analyses, confirm that the following items are present in the figure legend, table legend, main text, or Methods section.

n/a	Confirmed	
☐	☒	The exact sample size (*n*) for each experimental group/condition, given as a discrete number and unit of measurement
☐	☒	A statement on whether measurements were taken from distinct samples or whether the same sample was measured repeatedly
☐	☒	The statistical test(s) used AND whether they are one- or two-sided<br>*Only common tests should be described solely by name; describe more complex techniques in the Methods section.*
☐	☒	A description of all covariates tested
☒	☐	A description of any assumptions or corrections, such as tests of normality and adjustment for multiple comparisons
☐	☒	A full description of the statistical parameters including central tendency (e.g. means) or other basic estimates (e.g. regression coefficient) AND variation (e.g. standard deviation) or associated estimates of uncertainty (e.g. confidence intervals)
☐	☒	For null hypothesis testing, the test statistic (e.g. *F*, *t*, *r*) with confidence intervals, effect sizes, degrees of freedom and *P* value noted<br>*Give P values as exact values whenever suitable.*
☒	☐	For Bayesian analysis, information on the choice of priors and Markov chain Monte Carlo settings
☒	☐	For hierarchical and complex designs, identification of the appropriate level for tests and full reporting of outcomes
☐	☒	Estimates of effect sizes (e.g. Cohen's *d*, Pearson's *r*), indicating how they were calculated

Our web collection on statistics for biologists contains articles on many of the points above.

Software and code

Policy information about availability of computer code

Data collection	Data collection was done using open source software from miniscope.org (2018 version) allowing simultaneous data collection from an implanted miniscope and a behavioural camera
Data analysis	Data preprocessing was done using MATLAB (MathWorks, v. R2015a). Most of the data analysis was done using MATLAB (MathWorks, v. R2018b) a few exceptions include Python scripts run on PyCharm (v. 2018.3 (Edu)) with their outputs transferred to MATLAB. Open source code was used for cell segmentation (Miniscope software package (from miniscope.org (2018 version)); MATLAB (MathWorks, v. R2015a)), motion correction (Miniscope software package (Miniscope software package (from miniscope.org (2018 version)); MATLAB (MathWorks, v. R2015a)), spike inference (Miniscope software package (from miniscope.org (2018 version)); MATLAB (MathWorks, v. R2015a)) and head direction decoding (Python (PyCharm v. 2018.3 (Edu))). The remaining analysis was done using custom code written in MATLAB (MathWorks, v. R2015a)) except for the dimensionality reduction which was done using Python (PyCharm v. 2018.3 (Edu)) script that transfers the output to MATLAB.

For manuscripts utilizing custom algorithms or software that are central to the research but not yet described in published literature, software must be made available to editors and reviewers. We strongly encourage code deposition in a community repository (e.g. GitHub). See the Nature Portfolio guidelines for submitting code & software for further information.

Data

Policy information about availability of data

All manuscripts must include a data availability statement. This statement should provide the following information, where applicable:

- Accession codes, unique identifiers, or web links for publicly available datasets
- A description of any restrictions on data availability
- For clinical datasets or third party data, please ensure that the statement adheres to our policy

All data used in this manuscript will be made publicly available via: DIO:10.6084/m9.figshare.21792689

Field-specific reporting

Please select the one below that is the best fit for your research. If you are not sure, read the appropriate sections before making your selection.

☒ Life sciences ☐ Behavioural & social sciences ☐ Ecological, evolutionary & environmental sciences

For a reference copy of the document with all sections, see nature.com/documents/nr-reporting-summary-flat.pdf

Life sciences study design

All studies must disclose on these points even when the disclosure is negative.

Sample size	The number of subjects in this study was limited by the difficulty of accessing the mouse anterodorsal thalamic nucleus using miniaturized microscopes combined with thin (0.5mm diameter) relay lenses. The success rate of the surgeries being very low, our objective was to record from at least three mice per experiment.<br>On the other hand, for each mouse we get an average of over a hundred neurons simultaneously recorded per session which constitutes a sample size with an order of magnitude higher than the largest neural population ever reported in the studies of the head direction system, at the time of submission of this manuscript.
Data exclusions	Outliers were identified as data points outside the range of mean +/- 3*(standard deviation). If found, these outliers were excluded from the analysis.<br>Data automatically included based on specific selection criteria was manually checked to ensure inclusion was not biased by measurement noise.
Replication	Findings in this manuscript have been replicated in all animals and data per animal is shown in supplementary figures.
Randomization	This is not relevant to our study. We have a single experimental group.
Blinding	This is not relevant to our study. We have a single experimental group.

Reporting for specific materials, systems and methods

We require information from authors about some types of materials, experimental systems and methods used in many studies. Here, indicate whether each material, system or method listed is relevant to your study. If you are not sure if a list item applies to your research, read the appropriate section before selecting a response.

Materials & experimental systems

n/a	Involved in the study	
☒	☐	Antibodies
☒	☐	Eukaryotic cell lines
☒	☐	Palaeontology and archaeology
☐	☒	Animals and other organisms
☒	☐	Human research participants
☒	☐	Clinical data
☒	☐	Dual use research of concern

Methods

n/a	Involved in the study	
☒	☐	ChIP-seq
☒	☐	Flow cytometry
☒	☐	MRI-based neuroimaging

Animals and other organisms

Policy information about studies involving animals; ARRIVE guidelines recommended for reporting animal research

Laboratory animals	All animals used in this study are male wild-type mice (C57Bl/6, Charles River) aged between 6 and 8 weeks at the time of the first surgery.

Wild animals	The study did not involve wild animals.
Field-collected samples	The study did not involve field collected samples.
Ethics oversight	All experiments were carried out in accordance with McGill University and Douglas Hospital Research Centre Animal Use and Care Committee (protocol #2015-7725) and in accordance with Canadian Institutes of Health Research guidelines.

Note that full information on the approval of the study protocol must also be provided in the manuscript.
